# Healthcare system intervention for safer use of medicines in elderly patients in primary care—a qualitative study of the participants’ perceptions of self-assessment, peer review, feedback and agreement for change

**DOI:** 10.1186/s12875-015-0334-6

**Published:** 2015-09-04

**Authors:** Cecilia Lenander, Åsa Bondesson, Patrik Midlöv, Nina Viberg

**Affiliations:** Department of Clinical Sciences in Malmö, Lund University, Jan Waldenströms gata 35, SE-205 02 Malmö, Sweden; Department of Medicines Management and Informatics, Region Skåne, Sweden; Department for Public Health Sciences, Karolinska Institutet, Stockholm, Sweden

## Abstract

**Background:**

The elderly population is increasing and with advanced age comes a higher risk for contracting diseases and excessive medicine use. Polypharmacy can lead to drug-related problems and an increased need of health care. More needs to be done to help overcome these problems. In order for new models to be successful and possible to implement in health care they have to be accepted by caregivers. The aim of this study was to evaluate participants’ perceptions of the SÄKLÄK project, which aims to enhance medication safety, especially for elderly patients, in primary care.

**Methods:**

This is a qualitative study within the SÄKLÄK project. The SÄKLÄK project is a multi-professional intervention in primary care consisting of self-assessment, peer review, feedback and written agreements for change. A total of 17 participants from the intervention’s primary care units were interviewed. Most of the interviews were done on a one-to-one basis. The interviews were recorded and transcribed verbatim. A survey was also sent to the primary care unit heads. Qualitative content analysis was used to explore the participants’ perceptions.

**Results:**

The analysis of the interviews yielded six categories: *multi-professional co-operation*, *a focus on areas of improvement*, *the joy of sharing knowledge*, *disappointment with the focus of the feedback*, *spend time to save time and impact on work*. From these categories a theme developed: “Medication safety is a large area. In order to make improvements time needs to be invested and different professions must contribute.”

**Conclusions:**

This study shows that our studied intervention method is feasible to use in primary care and that the multi-professional approach was perceived as being very positive by the participants. Multi-professional co-operation was time consuming, but was also deemed as an investment and an opportunity to share knowledge. Some points of improvement of the method were identified such as simplification of the self-assessment form and clearer instructions for reviewers. In addition, to have an impact on work the focus must lie in areas within the primary care units’ scope.

**Electronic supplementary material:**

The online version of this article (doi:10.1186/s12875-015-0334-6) contains supplementary material, which is available to authorized users.

## Background

The elderly population is increasing worldwide and statistical demographic data suggest that ~22 % of the global population will be older than 65 years of age by 2050 [1]. In Sweden, the proportion of the population aged 65 years or older was 19.4 % in 2013 [2]. Ageing is known to be associated with an increased prevalence of multiple chronic diseases and as a result the use of more medications. Elderly patients with multiple diseases and polypharmacy risk suffering from drug-related problems. Previous studies have found that a significant proportion of hospital admissions among elderly people are due to adverse drug events (ADEs) [3–7]. Indicators of prescribing quality for drug treatment in the elderly have been developed in Sweden [8], as is the case in other countries [9] Elderly patients with multiple diseases and polypharmacy often have several prescribers. With many different systems for documentation, there is a big risk of medication errors, especially when these elderly patients are transferred from, for example, hospital care to primary care [10, 11]. General practitioners (GPs) are central to this work since they often have overall responsibility for these patients. If they do not have information about current drug use and take it in to account when prescribing, the risk of ADEs increases and compliance can decrease. Noncompliance can increase morbidity and thereby increase health care utilisation [12–14]. Different approaches to overcoming these problems have been tried [15], but more must be done in terms of, for example, co-operation between primary care and municipally provided home care [16]. A multidisciplinary approach to managing polypharmacy has been recommended in other countries, such as the United Kingdom [17]. No single intervention will solve all problems. Multiple interventions are needed instead [18].

The present study aimed to elucidate participants’ perceptions of the SÄKLÄK project, an intervention model created to improve medication safety for elderly patients in primary care.

The intervention model was originally developed for, and successfully implemented in, hospital care, to prevent birth injuries [19]. The model was then tested in orthopaedic surgery (PRISS) [20] and also in an ongoing project to improve abdominal surgery. For an intervention to be successful and possible to implement in health care it has to be accepted by the health care staff. The rationale for performing the entire SÄKLÄK project was to see if an improvement methodology, i.e. internal quality monitoring followed by external audit/peer review, can be applied in different settings. The intervention was adapted to primary care by the participating professional organisations (The Swedish College of General Practice, The Swedish Pharmaceutical Society, Geriatric Medicine in Sweden, Riksföreningen för Medicinskt Ansvariga Sjuksköterskor (a Swedish association of authorised nurses), Sweden’s National Organisation of District Nurses and The Swedish Society of Clinical Pharmacology and Therapeutics) and consists of self-assessment followed by peer review, feedback and a written agreement for change.

The aim of this study was to elucidate how the participants perceived a multi-professional intervention consisting of self-assessment, peer review, feedback and agreement for change.

## Methods

We did a qualitative study based on individual, semi-structured interviews supplemented with a survey. The interviews were analysed by manifest and latent qualitative content analysis to derive the participants’ experiences of a multi-professional project to enhance medication safety in elderly patients. The results from the interviews were triangulated with the survey responses.

### Setting

The interviews were performed with participants in the intervention group of the SÄKLÄK project. The survey was sent to the managers at the five intervention primary care units.

### Intervention model (SÄKLÄK project)

The SÄKLÄK project was initiated by the Swedish Association of Local Authorities and Regions (SALAR) and The Patient Insurance LÖF. The steering committee of the project consisted of one delegate each from six professional organisations (The Swedish College of General Practice, The Swedish Pharmaceutical Society, Geriatric Medicine in Sweden, Riksföreningen för Medicinskt Ansvariga Sjuksköterskor (a Swedish association of authorised nurses), Sweden’s National Organisation of District Nurses and The Swedish Society of Clinical Pharmacology and Therapeutics). The SÄKLÄK project was a pilot study to determine whether an intervention model (see Table 1), developed in hospital care, could be used in primary care to enhance medication safety in elderly patients. Based on previous studies [19, 20] it was concluded that self-assessment was valuable, that external peer reviews prevent postponing of the self-assessment and that the review process supports ongoing improvement and encourages new improvement projects. It was also noted that reviewers learn a lot, become aware of patient safety risks in their clinics and bring improvement ideas back to their clinics. However, the reviewers need to receive clear instructions to focus on achievable goals.

**Table 1:** Description of the different parts of the tested intervention model (SÄKLÄK)

1. Introductory meeting	Representatives from the steering committee^a^ visited the primary care units, gave a structured introduction and presented the intervention model for unit managers and staff representatives, including nurses working in home care and pharmacists. The involvement of all professional categories was presented as a prerequisite for the self-assessment process.
2. Structured self-assessment	The self-assessment was developed by an expert group, appointed by the steering committee^a^. It contained 12 questions covering areas of importance for safe use of medications in primary care, with focus on elderly patients with multiple diseases. The areas covered were: prescribing of drugs, follow-up, medication reviews, environmental aspects, co-operation with specialized care, pharmacies and communal home care. For each of the 12 questions, five follow-up questions were asked:
	1. What methods/routines/guidelines do you have?
	2. How do you provide conditions to ensure compliance?
	3. How do you measure compliance?
	4. How do you give feedback on the results to the staff?
	5. What ideas do you have for improvement?
3. Peer review	A group of doctors, nurses and pharmacists selected by the professional organisations^b^ served as reviewers. For each primary care unit, a peer-review team consisting of five to six reviewers with different professions was formed at a seminar 4 months after the project was initiated. At this meeting the teams discussed the answered self-assessments and how to conduct the site visits. The primary care units were visited by a peer-review team 5 months after the project was initiated. A document based on the questions used in the self-assessment procedure served as support for the peer review. New or updated information arising during the visit or in dialogue with the primary care unit was noted in this document.
4. Written feedback and agreement for change	The peer-review team presented a written feedback report regarding their view on strengths and weaknesses, priority areas for improvement and proposed measures to be taken. Eventually, a written contract consisting of a detailed action plan was jointly produced by the primary care unit and the peer-review team.
5. Follow-up seminar	A seminar for the steering committee, the reviewers and all managers at the intervention primary care units.
6. Follow up on accomplishment of agreements	The agreements for change were to be followed up on 6 months after they were signed.

^a^The steering committee comprised representatives from the Swedish Association of Local Authorities and Regions (SALAR) and The Patient Insurance LÖF and one delegate each from the six professional organisations listed below

^b^The Swedish College of General Practice, The Swedish Pharmaceutical Society, Geriatric Medicine in Sweden, Riksföreningen för Medicinskt Ansvariga Sjuksköterskor (a Swedish association of authorized nurses), Sweden’s National Organisation of District Nurses and The Swedish Society of Clinical Pharmacology and Therapeutics

The aim of the SÄKLÄK project was to reduce medication errors and drug-related problems.

The first step of the intervention was a self-assessment questionnaire, with questions on how patient safety is secured during prescribing of medication, medication use and follow up, at the primary care centre and in cooperation with pharmacies/hospitals/municipally provided home care. The questions were focused on frail elderly people, on how conditions are to be provided for different measures of importance and, not least, how it is ensured that these measures are being followed. A group of selected doctors, nurses and pharmacists, with vast experience in elderly care, served as reviewers in the second step of the intervention. The assessment by the reviewers comprised of:

- Receiving and analysing of self-assessment forms, and discussion of these
- Visits to primary care units with opportunities to ask questions and share their views
- Feedback and agreement for change

**Fig. 1:**
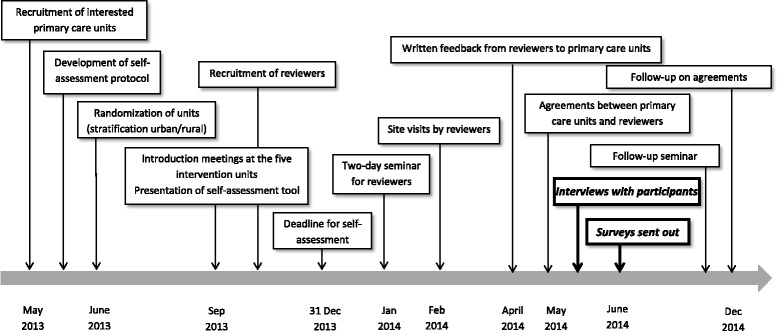
Overview of the time schedule for the SÄKLÄK project. Areas covered in this article in bold

Supported by written instructions, documents and continuous contact with the project management the reviewers analysed the self-assessments and any additional material supplied by the primary care units. They had the possibility to get clarifications on their questions during site visits. Thereafter the reviewers produced a written feedback report for the primary care unit, and the reviewers and the management at the primary care unit agreed on an action plan for improvements. The procedures of the intervention model are described in Table 1 and Fig. 1. Invitations to participate were emailed to all primary care units in Sweden (approximately 1200) and participation was open to all. A total of 20 units applied and they were stratified according to urban or rural location. A random sample of 10 units was drawn using Excel. Five units were randomised to the intervention group and five to the control group, keeping the distribution between urban and rural units. The control units were recruited for later comparison on quantitative data (not yet available).

### Interviews

At the five intervention primary care centres, the managers were asked to identify individuals who had had an active role in answering the self-assessment questions and to invite them to be interviewed. A total of 17 persons participated in 15 interviews (two interviews were held with two subjects together). Furthermore, 13 of the interviews were performed face-to-face at the primary care centres and two were conducted via telephone (see Table 2 for more information).

**Table 2:** Presentation of the interviewees

Occupation	Years in current position	Gender	Interview	Location
General practitioner	17	Female	Face-to-face	Urban
Head of primary care centre	1	Female	Face-to-face	Urban
District nurse	30	Female	Face-to-face	Urban
District nurse working in municipally provided home care	6	Female	Face-to-face	Urban
Pharmacist working at a pharmacy	20	Female	Face-to-face	Urban
Pharmacist working at primary care centre	1	Female	Face-to-face	Urban
General practitioner	14	Male	Face-to-face	Urban
District nurse	42	Female	Face-to-face	Urban
General practitioner	25	Male	Face-to-face	Urban
Head of primary care centre	4.5	Female	Face-to-face	Urban
Two district nurses working in municipally provided home care	4	Females	Face-to-face	Urban
Head of primary care centre	4.5	Female	Face-to-face	Rural
District nurse	3	Female	Face-to-face	Rural
MAS^a^ (nurse)	12	Female	Telephone	Rural
Head of primary care centre	-	Female	Telephone	Urban
Administrator	-	Female		

^a^A nurse with responsibility for health care in the municipality

All interviews were performed by the first author (C.L.). The interviews were conducted with an interview guide (Additional file 1) and lasted for between 8 and 36 min. Questions were asked regarding, for example, positive and negative experiences of participating in the project, perceptions of the different steps of the project and collaboration between different professionals. The interviewer is a clinical pharmacist and has 15 years of experience working with elderly patients and their medication use at pharmacies, in hospital care and in primary care. Prior to the interview process the interviewer completed a course for doctoral students in qualitative methods, including interview techniques. One of the authors (NV) is experienced in interview studies and provided supervision. All interviews were recorded and transcribed verbatim.

### Analysis of interviews

The transcribed interviews were subjected to qualitative content analysis, a research method for interpreting the content of text data through systematic classification by coding and identifying themes or patterns [21]. The analysis was performed in several steps and was initiated before all interviews had been conducted. After reading the text several times to get a sense of the whole, the text was divided into meaning units. These meaning units could be words, sentences or paragraphs containing aspects related to each other through their content and context. The meaning units were condensed and thereafter coded. The codes were then sorted into categories. A deeper analysis was thereafter performed to find the latent meaning of the interviews, giving a theme [22]. The analysis started as soon as the first interview was transcribed. Thereafter the analysis ran parallel to the interviews and after 15 interviews saturation was reached. The codes and categories were not predefined, but instead developed during the analysis (Table 3). To enhance trustworthiness, the codes and categories were discussed within the research group throughout the analysis process. The findings are illustrated by citations from the interviews to show that the findings derive from the data. The citations have been translated from Swedish. All citations can be tracked by a letter identifying the interviewed individual, and a number indicating the line in the transcribed interview.

**Table 3:** Example of how findings were yielded from the analytical process, showing some of the many meaning units that built up the categories and the theme

Meaning unit	Condensed meaning unit	Code	Category	Theme
To see all this and to listen to other people participating, to listen to their ways of seeing things	Listen to other people’s way of seeing things	Co-operation	Multi-professional co-operation	Medication safety is a large area. In order to make improvements time needs to be invested and different professions must contribute
I cannot make progress in this matter, but is there someone else who has managed it, and if so, how?	Helping each other	Knowledge sharing	The joy of sharing knowledge	
It was quite comprehensive, so it took a lot of time to go through it all and answer all the questions	Comprehensive form and time-consuming to answer	Time-consuming	Spend time to save time	
It costs energy right now, but if we can improve our routines and follow them… then I think it will pay off in the long run	Investing time now will save time later	Time-saving		
Not that I clearly felt that we were talking about the answers in our self-assessment report	Not talking about the same things	Disappointment	Disappointment with the focus of the feedback	
I think it was really good to see it in print, what’s working and what’s not, and what we can improve	What’s working and what’s not, and improvements	Strengths and weaknesses	A focus on areas of improvement	
Yes, concerning medication reviews I think so […] we can surely perform many more of these	Perform many more of these	Change of routines	Impact on work	

### Surveys

At the end of the SÄKLÄK project a survey was sent to the heads of each of the five intervention primary care units. The survey was a web questionnaire and contained questions about how the heads perceived the initial information regarding the project, the different components of the project and the support available. It included both multiple choice and open-ended questions (Additional file 2). The survey was based on the survey questions used in the birth injury-project [19] in order to make a comparison between the projects possible. The survey responses were carefully read and compared with the findings from the interviews.

### Validation

The results of the qualitative analysis were reported to the managers, the reviewers representing different professional organisations, and the SÄKLÄK project steering committee at a follow-up seminar.

### Ethical considerations

Approval was granted by the Research Ethics Review Board in Lund (reference no. 2013/333). Participation in the study was based on informed consent. Consent to publish was obtained from all interviewees (Table 2).

## Results

Based on identified categories a theme emerged: “Medication safety is a large area. In order to make improvements time needs to be invested and different professions must contribute.” (Table 3).

### Interviews

The qualitative content analysis of the interviews yielded six categories.

### Multi-professional co-operation

The participants noted the project’s multi-professional approach as something very positive. It was an opportunity to invite people from the pharmacy, the hospital and municipal home care to engage in closer co-operation, to meet face to face.

As one respondent said:

*“…the most positive part has been working with the self-assessment, working multi-professionally and getting a better understanding of each other’s work.” (primary care unit head, C4)*

The multi-professional approach seems to have given the participants a chance to meet in person and discuss questions of importance for medication safety. Many of them said that they had been working “together” for a long time, but now realised it was more side-by-side than real co-operation.

*“…I have thought one step further: we need to help each other…” (nurse, E40)*

### A focus on areas of improvement

According to the participants, the self-assessment process highlighted specific areas for improvement, but also showed strengths of the primary care centre. Examples of identified areas for improvement were: keeping an accurate medication list, factors affecting the prescribers’ choice of therapy and factors affecting patients’ ability to contribute to drug safety. Identified strengths included: committed leadership, climate open to discussion, existing routines and access to consultants (geriatricians, psychiatrists, pharmacists). Areas for improvement were also highlighted in the written feedback, which was appreciated by the participants. One person pointed out that many questions in the self-assessment concerned monitoring, and that monitoring a lot of things could distract from the aim to increase patient safety.

*“…seeing what we have done and what we need to improve, presented in a clear and concise way, is positive.” (primary care unit head, U15)*

*“I look at the medicines in another way now.” (nurse, E7)*

### The joy of sharing knowledge

The visits by the peer review teams were viewed as mostly enjoyable and exciting, with no feeling of being investigated. The group of reviewers, with working experience from different areas, were perceived as being able to bring a lot of knowledge to the primary care centre, but also to learn some new things to take home.

*“…it’s great to have a peer review team from different parts of the country with different viewpoints. […] Sometimes they said this is not how we do it, but you seem to have found a good solution…” (primary care unit head, J112)*

*“…they were interested and had questions. We had a good discussion…” (primary care unit head, V11)*

### Disappointment with the focus of the feedback

Some respondents expressed slight disappointment that the peer review visits sometimes lacked a summary at the end of the day. They had hoped for a short summary of their strengths and weaknesses. Many of the participants felt that the written feedback did not always focus on the primary care unit; it was sometimes more of a discussion at a higher level.

*”…some feedback at the end of the visit. But I didn’t get that with me, it was more of a general discussion of Swedish health care…” (primary care unit head, C14)*

*”…these are things outside our influence.” (GP, B106)*

### Spend time to save time

The project took more time than expected, according to the participants. Finding interested participants outside the primary care centre and explaining the aim of the project to them was more time-consuming than expected. The self-assessment form was felt to be too long and filling it out online took a lot of time.

*“…to include people outside the primary care unit took a lot of time” (primary care unit head, U4)*

*”It took longer than I had initially expected” (primary care unit head, L9)*

On the other hand, some of the participants pointed out that the time invested in the short-term may save time later on through improved routines for safe use of medications.

*“…it costs energy right now, but if we can improve our routines and follow these […] then I think it will pay off in the long run…” (GP, G117)*

### Impact on work

The participants viewed written agreements as something positive – a reminder to keep focused on medication safety – but also perceived it as a little stressful. The follow-up was six months later and summer was coming in between. At some primary care units the self-assessment led to a change of routines right away, while others stated that changes would be made in the future.

*“Yes, it has already [started to yield change]…” (GP, B167)*

*“It can only get better. More structure, get more routines…” (nurse, H62)*

### Surveys

All five managers completed the survey. Four of them also participated in the interviews. The responses from the surveys concur in general with the interviews. The respondents pointed out the multi-professional approach as being very positive. The self-assessment was said to be worthwhile to identify strengths and weaknesses. According to one respondent this was the only useful part of the project, even though it took more time than expected. At one of the participating primary care units they felt criticised by the reviewers, according to the survey. This did not come up during the interviews.

### Validation

The results of the qualitative analysis of the interviews were discussed at the follow-up seminar. The participants agreed with the findings.

## Discussion

One of the most positive experiences of the intervention, according to the participants, was the focus on multi-professional co-operation. It seemed as though the project opened the eyes of the participants to the importance of working together on the big issue of medication safety. Other ways to improve medication safety in the elderly population, such as medication reviews and medication reports, involve multiple professions and have been shown to reduce drug-related problems [10, 17, 23]. Still, the care of the elderly remains fragmented [18] and co-operation between all health care professionals involved in the care of the elderly needs to be improved. This is something this method might contribute to, since the multi-professional approach was pointed out as being something very positive by the participants.

The self-assessment was an appreciated and useful tool to identify areas of improvement for medication safety at primary care units and to clarify strengths and weaknesses. Other studies, both in the health care system and in other areas, have shown similar results [19, 24]. It is also important to involve both management and employees for the self-assessment to achieve acceptance and success [24]. The self-assessment in the SÄKLÄK project was constructed in such a way that no person could answer all questions by himself or herself, but had to include other health professionals. And since there’s more to collaboration than simply working side by side as health professionals [25], the self-assessment may serve as a tool to facilitate communication between different professionals. The British National Institute for Health and Care Excellence (NICE) has acknowledged that “no-one who works alone can stay at the forefront of knowledge given the speed of organisational and clinical change”.

The focus of the feedback was sometimes on general problems rather than specific problems at the primary care unit, which disappointed many participants. The reviewers were criticised for proposing solutions that were not applicable. These problems were also seen when testing the method at Swedish maternity units [19], despite instructions for the reviewers involved to be humble and to have realistic expectations on feasible measures of actions within the healthcare system. These aspects raise questions about how to use the peer review process in other interventions, and how peer reviewers should be selected, trained and instructed. Peer review can be explained as knowledge sharing [26] and is not only useful for the reviewed unit but also for the reviewers. The sharing of knowledge was highlighted as a positive part of this project by some participants. Peer review has been shown by others to be useful to shed light on provider-related errors and associated safety concerns, some of which may be modifiable [27]. This is of course useful for a single primary care unit, but also at an aggregated level to see patterns at, for example, the national level [27]. In this study all units got suggestions in their written feedback that they felt was at a higher level and not within their scope. These suggestions will be analysed and used to improve the health care system.

The participants perceived some parts of the intervention, i.e. self-assessment, as time-consuming. In a pilot study like this, finding out what works and what does not is part of the evaluation. On the other hand, according to some participants the time invested in the short-term may be recouped later in form of, for example, better routines. Almost all respondents felt that participating in this project would affect their work, and thereby were in support of efforts to improve medication safety in the elderly. One interviewee expressed some annoyance over the fact that many of the questions in the self-assessment included monitoring. This could take focus from more important things, for example improving medications safety, according to the interviewee. It is important to select variables focusing on medication safety if monitoring should be a part of the routines.

This interview study indicates that a program consisting of self-assessment, peer review and feedback can be a valued tool to assess patient safety. This is supported by the findings of Meeks et al. [27], who found that health care organisations could renew their peer review programs to enable self-assessment, feedback and improvement and thereby increase patient safety.

Participation in this project was not mandatory; rather, it was to be seen as an opportunity to improve medication safety. Most of the participants seemed to agree that primary care units have a responsibility to help solve the problems concerning medication safety. But, again, focus in the feedback from reviewers must be on goals within the primary care units’ scope.

### Methodological discussion

As the design is qualitative it should be assessed by means of trustworthiness, which includes credibility, transferability and dependability. Credibility deals with the focus of the research and refers to confidence in how well data and analytical processes address the intended focus [22]. The participants represented different professions within Swedish primary care and had varying working experience. All but one received their medical education in Sweden. The interviews resulted in large amounts of material. We performed 15 interviews with 17 participants, and the parallel analysis showed that saturation was reached in the data. The number of participants interviewed varied between primary care units, with two units providing the majority of interviewees. This may have influenced the results since these units were very engaged in the project and both had an urban location. To improve trustworthiness, the results from the interviews and from the surveys were triangulated. This process revealed that one primary care unit felt questioned by the reviewers, which was not mentioned during the interviews. We also reported the findings from the qualitative analysis back to the participants to see if they agreed, which they did. To increase credibility, the analysis was discussed within the research group and the analytical process is shown in Table 3. Illustrative quotations from the interviews have been provided to show that the categories come from the data.

The original plan was to conduct the interviews after all units had completed every step of the intervention. However, due to an unexpected delay in providing written feedback to some units a few of the interviews were performed before the agreements for change had been signed. This might have affected the results. However, the surveys were sent out after the agreements had been signed and the answers concur with the interviews.

Dependability concerns the degree to which data change over time and alterations made in the researcher’s decisions during the analysis process. Data were collected using a semi-structured interview guide, and according to Graneheim and Lundman [22] this can strengthen trustworthiness. The guide ensured that all participants were asked the same questions.

Transferability refers to the extent to which the findings can be transferred to other settings and groups, and it is up to the reader to judge it [22]. We elucidated the participants’ perceptions of a multi-professional intervention model to enhance medication safety in primary care. A potential limitation is that although participation was open to all primary care units in Sweden, we do not know if the selected units are an accurate representation of Swedish primary care. A total of 20, out of the 1200 possible primary care units in Sweden, applied for participation in the study. These health care units were from different parts of the country in both urban and rural locations. However, this was a qualitative evaluation of a pilot study and the aim was to explore the perceptions of the method from the participants. A strength of this study is the use of the same interviewer for each interview, which meant there was no need to calibrate answers from different interviewers.

This research group has extensive experience from health care, especially elderly and medications. CL is a pharmacist and PhD-student, ÅB a pharmacist and PhD, PM an MD and associate professor, NV is a pharmacist and PhD.

### Future research

This method needs further development. Potential modifications can be made based on the results of this pilot study. These include simplifying the self-assessment process and offering clearer instructions to reviewers and primary care management. It would also be beneficial for more primary care units to be given the opportunity to participate in similar studies in the future. Different models for improving medication safety in the elderly population need to be compared, and for these models to be successful we must know that the participants accept, or even welcome, the intervention.

## Conclusion

This study shows that our studied intervention method is feasible to use in primary care and that the multi-professional approach was perceived as being very positive by the participants. Multi-professional co-operation was time consuming, but was also deemed as an investment and an opportunity to share knowledge. Some points of improvement of the method were identified, such as simplification of the self-assessment forms and clearer instructions for reviewers. Furthermore, in order to have an impact on work the focus must lie in areas within the primary care units’ scope.

## Additional files

Additional file 1:
**Interview guide.** (DOCX 14 kb) Additional file 2:
**Survey to primary care centres.** (DOCX 17 kb)
