# Supplementary material for: Healthcare system intervention for safer use of medicines in elderly patients in primary care—a qualitative study of the participants’ perceptions of self-assessment, peer review, feedback and agreement for change
Source: BMC Fam Pract. 2015 Sep 4;16:117. doi: 10.1186/s12875-015-0334-6 (PMC4559262; doi:10.1186/s12875-015-0334-6)
Supplement: Additional file 1: — Interview guide. (DOCX 14 kb) [file 12875_2015_334_MOESM1_ESM.docx]

**Appendix 1. Interview guide.**

1. Tell me something positive about participating in this project?
2. Tell me something negative about participating in this project?
3. How did you perceive the four steps of the process?
   - Self-assessment
   - Peer-review
   - Written feedback
   - Agreement for change
4. How did you perceive the co-operation with other professionals and staff?
5. How do you think participation in this project will affect work at your primary care centre?
6. If you had the power to decide, how would you organize the care for frail elderly with many diseases?
7. Do you have anything else you would like to add?

Each question was followed by exploring questions, for example “Can you tell me more about that?” or “Can you give me an example?”
