# Supplementary material for: Healthcare system intervention for safer use of medicines in elderly patients in primary care—a qualitative study of the participants’ perceptions of self-assessment, peer review, feedback and agreement for change
Source: BMC Fam Pract. 2015 Sep 4;16:117. doi: 10.1186/s12875-015-0334-6 (PMC4559262; doi:10.1186/s12875-015-0334-6)
Supplement: Additional file 2: — Survey to primary care centres. (DOCX 17 kb) [file 12875_2015_334_MOESM2_ESM.docx]

**Appendix 2. Survey to primary care centres**

1. **Introduction meeting at the primary care centre**

The information we received at the introduction meeting was sufficient.

Please grade the information provided at the introduction meeting, using a scale of 0-7 (7 being ‘excellent’, 0 being ‘insufficient’). Please provide additional comments and suggestions in the box below*.*

1. **The self-assessment tool**

The self-assessment tool worked satisfactorily to identify strengths and areas of improvement.

Please grade the tool, using a scale of 0-7 (7 being ‘excellent’, 0 being ‘insufficient’). Additionally we’d love to hear what strengths and weaknesses you noticed in the tool, if anything was missing, or redundant, or difficult to understand.

Please provide additional comments and suggestions in the box below.

1. **The self-assessment report**

Our self-assessment report (including accompanying appendices) gave us a sufficient tool for identifying strengths and areas of improvements.

Please grade the tool, using a scale of 0-7 (7 being ‘excellent’, 0 being ‘insufficient’). Please provide your additional comments and suggestions in the box below.

1. **The site visit**
2. The reviewers’ site visit was perceived as positive by the co-workers interviewed.

Please grade the statement above, using a scale of 0-7 (7 being ‘completely agree’, 0 being ‘do not agree at all’). Please provide your additional comments and suggestions in the box below.

1. My experience was that we had a good and open dialogue with the reviewers.

Please grade the dialogue using a scale of 0-7 (7 being ‘completely agree’, 0 being ‘do not agree at all’). Please provide additional comments and suggestions in the box below.

1. **The feed-back**
2. The reviewers were clear with the deficiencies in patient safety they identified.

Please grade the statement above using a scale of 0-7 (7 being ‘completely agree’, 0 being ‘do not agree at all’).

1. The reviewers pointed out deficiencies in patient safety we were not aware of.

Please grade the statement above using a scale of 0-7 (7 being ‘completely agree’, 0 being ‘do not agree at all’).

1. We appreciated the feedback from the reviewers.

Please grade the statement above using a scale of 0-7 (7 being ‘completely agree’, 0 being ‘do not agree at all’). Please provide additional comments and suggestions in the box below.

1. **The web application**

The web application worked satisfactorily.

Please grade the web application, using a scale of 0-7 (7 being ‘excellent’, 0 being ‘insufficient’). Please provide additional comments and suggestions in the box below.

1. **The Agreement for change**

We easily agreed with the reviewers on the content of the Agreement of change*.*

Please grade the statement above using a scale of 0-7 (7 being ‘completely agree’, 0 being ‘do not agree at all’). Please provide additional comments and suggestions in the box below.

1. **Ownership**

An important basis for the project was the primary care centre’s ownership for both the problems and the opportunity and responsibility to correct them. To what extent did the review and follow-up convey this goal?

Please grade the process using a scale of 0-7 (7 being ‘completely agree’, 0 being ‘do not agree at all’). Please provide additional comments and suggestions in the box below.

1. **The project administration**

I’m satisfied with the service of the project administration.

Please grade the project administration, using a scale of 0-7 (7 being ‘excellent’, 0 being ‘insufficient’). Please provide additional comments and suggestions in the box below.

1. **Templates for checklists, PMs etcetera**

Do you think it would be a good idea to produce templates for checklists or memos in certain areas relating to the safe use of medications in primary care? (yes/no)

If yes, which areas should be prioritized?

1. **General reflections on the project**
